# Interplay between Polymorphism and Isostructurality in the 2-Fur- and 2-Thenaldehyde Semi- and Thiosemicarbazones

**DOI:** 10.3390/molecules25040993

**Published:** 2020-02-23

**Authors:** Marcin Swiatkowski, Agata Trzesowska-Kruszynska, Agnieszka Danielewicz, Paulina Sobczak, Rafal Kruszynski

**Affiliations:** Institute of General and Ecological Chemistry, Lodz University of Technology, Zeromskiego 116, 90-924 Lodz, Poland

**Keywords:** polymorphism, isostructurality, thiosemicarbazone, semicarbazone

## Abstract

The four compounds, namely: 5-nitro-2-furaldehyde thiosemicarbazone (**1**); 5-nitro-2-thiophene thiosemicarbazone (**2**); 5-nitro-2-furaldehyde semicarbazone (**3**); and 5-nitro-2-thiophene semicarbazone (**4**) were synthesized and crystallized. The three new crystal structures of **1**, **2**, and **4** were determined and compared to three already known crystal structures of **3**. Additionally, two new polymorphic forms of **1** solvate were synthesized and studied. The influence of the exchange of 2-thiophene to 2-furaldehyde as well as thiosemicarbazone and semicarbazone on the self-assembly of supramolecular nets was elucidated and discussed in terms of the formed synthons and assemblies accompanied by Full Interaction Maps analysis. Changes in the strength of IR oscillators caused by the molecular and crystal packing effects are described and explained in terms of changes of electron density.

## 1. Introduction

Knowledge about non-covalent interactions increases with each passing year [1,2,3,4,5], but is still insufficient to enable the complete design and prediction of the crystal structure of organic compounds, even with the usage of computational crystal structure prediction. Application of computational methods allows for the prognostication of crystal packing on the basis of the structural formula, but these methods still lead to ambiguous results or give an incorrect build of the crystal net in some cases [6,7]. Through the years, crystal engineering principles have been developed on the basis of crystallographic data. The hydrogen bond rules [8,9], exchange rule [10,11], synthon hierarchy for some functional group [12,13,14,15,16], and molecular tectonics [17,18,19] are the most important principles among all of the studied ones. They are very helpful in predicting the results of crystal formation, but are still insufficient. Better understanding of the nature of intermolecular interactions and the ability to foresee the molecular structure are the key aspects for the engineering and control of crystal packing and, hence, the physical and chemical properties. 

The molecular geometry (including conformation and specific positions of functional group) as well as the intermolecular interactions existing between molecules have an influence on the crystal packing (the mutual arrangement of molecules in the crystal net). Molecular shape prediction is especially problematic for flexible molecules. It has been shown that similarly shaped molecules, not necessarily showing the chemical similarity, adopt the same packing arrangement [20]. Different conformers of the same molecules can have a different spatial arrangement of molecules [21]. Molecules do not always adopt the lowest energy conformer, which is balanced by the possibility of the formation of stabilizing inter- and intramolecular interactions. Some small changes in the molecular structure might result in significant changes in crystal packing. Nevertheless, specific functional groups can be interchanged without affecting the crystal structure. Such groups should not be involved in any hydrogen bond or electrostatic interactions [11]. Thus, the interplay between these two factors affects the arrangement of the molecules in the solid state. 

The presented study demonstrates the complexity of the problem of the self-assembly of different isomorphous molecules (i.e., 5-nitro-2-furaldehyde thiosemicarbazone (**1**), 5-nitro-2-thiophene thiosemicarbazone (**2**), 5-nitro-2-furaldehyde semicarbazone (**3**), 5-nitro-2-thiophene semicarbazone (**4**), Scheme S1). Only one compound of this group was previously structurally determined (i.e., the **3** [17,18,19]) and it created three monoclinic polymorphic forms **3*α*** [22], **3*β***, and **3*γ*** [23,24]. The introduction of additional species (different acids) to **3** distinctly affects the present synthons and hydrogen bonding schemes. The presence of additional hydrogen bond donors and acceptors and, more importantly, the presence of additional non-isomorphous species in the crystal net causes the impossibility of the formation of similar crystals [24]. The studied compounds are effectively isomolecular (i.e., have the same configuration (E) about the imine double bond, possess heteroatoms at the same sites of molecule and heteroatoms (O and S) [11], which possess the same electron configuration of the valence shell). As two heteroatoms are present in each molecule, a total of four combinations could be attained and consequently four compounds were studied. Such an approach allows a comparison of compounds with gently tuned molecular properties as well as the study of the slight expansion of the atomic core size on the self-assembly processes, in which very similar hydrogen-bonding groups compete during synthon formation. Additionally, in all four cases, the molecular space filling is practically the same, thus it has little influence on the crystal packing, and permits study of the impact of the interactions themselves (this is not possible in the case of studying compounds that possess other structurally dissimilar species in the crystal net [24]).

## 2. Results and Discussion

### 2.1. Synthesis 

The pure compounds **1**, **2**, and **4** were synthesized in only one polymorphic form (Table 1). Recrystallization from different solvents always led to the starting form in the case of compounds **2** and **3**. The use of dmf (pure or in mixture with other solvents) in the crystallization of **1** led to the formation of two polymorphic forms of dmf solvate of **1**. The 5-nitro-2-furaldehyde thiosemicarbazone dimethylformamide solvate **1·dmf*α*** was created during the slow evaporation of the dmf solution of **1**, while **1·dmf*β*** was crystallized from the MeOH:dmf (1:1 *v/v*) solvent mixture (Table 1). Exchanging MeOH for EtOH or *i*-PrOH in the mixture does not affect the final form, which suggests that the presence of the short chain alcohol is a necessary and sufficient condition for the formation of **1·dmf*β***. The crystals of compounds **2** and **4** are isostructural and these compounds did not create solvates or other pseudopolymorphs in the solid state, nevertheless of the solvents used during crystallization. It must be outlined that the application of very specific conditions such as high pressure can hypothetically lead to the formation of other polymorphic forms, but any evidence of the formation of other than the described forms was not registered during the study (including heating up of the compounds to their decomposition or cooling them to 100 K).

The polymorphic form **3*β*** was created as a result of the condensation reaction between 5-nitro-2-furaldehyde and semicarbazide hydrochloride carried out in water (in the presence of sulfuric acid) or in methanol (Table S1). The IR spectrum of this form matched the spectrum of the nitrofural pharmaceutical standard. The recrystallization of form **3*β*** from different solvents and solvent mixtures (dmf, *i*-PrOH, water, dmf:acetone (1:1 *v/v*), MeOH:water (1:1 *v/v*)) led to the starting form **3*β***. The usage of the MeOH:MeCN (1:1 *v/v*) solvent mixture or sole MeCN gave the polymorphic form **3*γ*** (Table S1). According to data presented in [23], the crystallization from MeOH:MeCN (3:1 *v/v*) or from A:B (1:3 *v/v*, where A is H_2_O, MeOH, EtOH, or butan-2-ol and B is MeCN or acetone) solvent mixture leads to the formation of **3*β*** and from the water:acetone (7:15 v/v) mixture or pure methanol, ethanol, propan1-ol, propan-2-ol, butan-2-ol, and acetonitrile leads to the formation of **3*γ***. These data are partially in opposition to the current findings and seem to be slightly internally inconsistent as an excess or shortage of the aprotic solvent in the mixture led to the formation of **3*β*** or **3*γ*** without any clear tendency, and the usage of pure solvent of any kind gave **3*γ***. The results of the current study suggest that the addition of an equivolume amount or volumetric excess (up to pure solvent) of MeCN gave the polymorphic form **3*γ***, while the other used pure solvents or solvent mixtures gave the polymorphic form **3*β***. The known polymorphic form **3*α*** [22] could not be obtained regardless of the crystallization conditions or synthesis method (application of the industrial method, which was the source of form **3*α*** described in [22] also led to form **3*β***). It can be suggested that form **3*β*** is predominant and form **3*α*** can be a “vanishing polymorph” (in subsequent works [23,24], the presence of form **3*α*** was also not registered). 

### 2.2. Molecular Conformation

**Figure 1:**
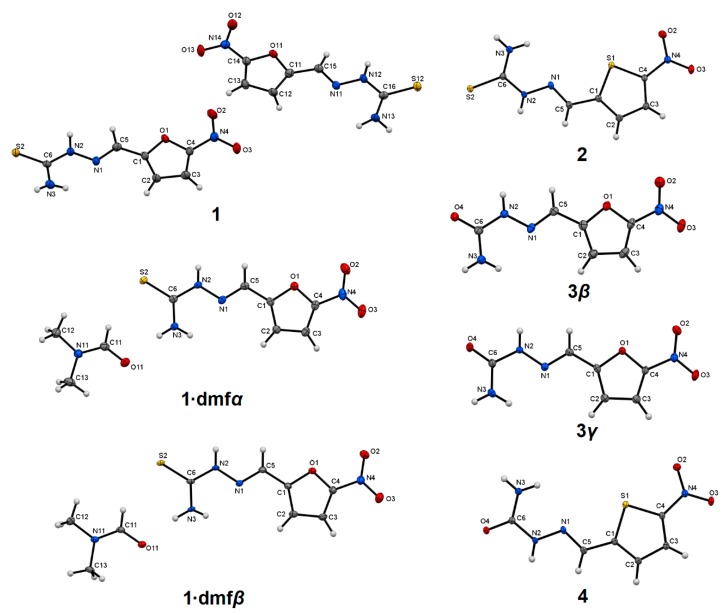
Solid state structures of the studied compounds with the atom numbering scheme, plotted with a 50% probability of displacement ellipsoids of non-hydrogen atoms. Hydrogen atoms are plotted as spheres of arbitrary radii.

**Figure 2:**
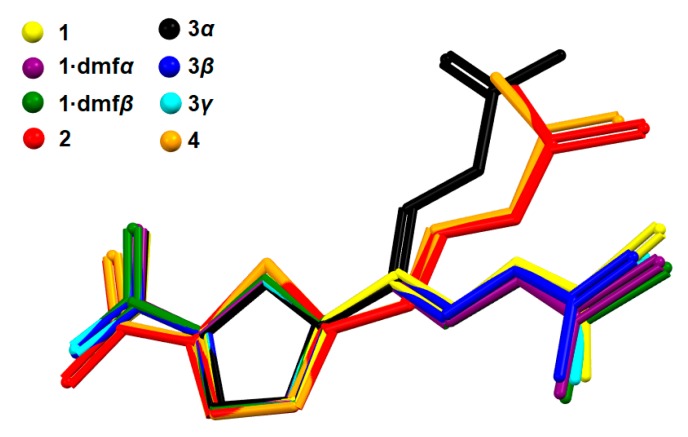
The molecular overlay of the studied compounds. The five-membered rings were arranged to provide the shortest distances between their constituent atoms.

The bond distances in all studied compounds (Figure 1) were similar (Table S2). To ensure that the temperature of the measurement did not affect the polymorphic form/stability, the structures of **3*β*** and **3*γ*** were redetermined at 100 K (temperature used in all other measurements of **1**–**4** structures; in previous studies the 173 K [24] and room temperature [22,23] were used). The compounds **3*β*** and **3*γ*** possessed almost identical structures at all temperatures and slight differences originated from normal changes of atom motion imposed by temperature. The molecules of thio- and semicarbazones adopted a nearly planar conformation with the *E* configuration about the imine C=N bond, but differed in arrangement of the heterocyclic ring with respect to the thio- and semicarbazone moiety and the orientation of the amide group. A superimposition of the studied compounds showed two groups of essentially different conformations, especially visible in the mutual arrangement of the five-membered ring and amide group (Figure 2). The first most populated arrangement is related to the possibly longest molecule (i.e., the aliphatic chain expanding along line perpendicular to the two-fold axis of five-membered ring). This conformation exits in **1**, **1·dmf*α***, **1·dmf*β***, **3*β***, and **3*γ***. The second, less populated conformation is adopted by isostructural compounds **2** and **4** as well as by **3*α***, but in the last case, the deviation from the above-mentioned longest molecule line was greater than for **2** and **4**. The values of O/S(ring)–C–C–N(imine) and N(imine)–N–C–N(amine) torsion angles (Table S2) confirm that nitrofuran containing compounds (**1**, **3*β*** and **3*γ***) possess different molecular conformation than nitrothiophene containing compounds (**2** and **4**). The **3*α***, which additionally shows a different orientation of the amide group, can be considered as belonging to a separate subgroup of the second general conformation. 

The energy barrier of rotation of the thio- and semicarbazone moiety about the C(imine)–C(ring) bond was much lower than the barrier of rotation of the thio- and amide group about the C(carbonyl)–N(H) bond (Figure S1). In both cases, the most unfavorable conformation was the one with an almost perpendicular orientation of the rotated group toward the plane of the ring. The larger energy barrier of rotation of the thio- and amide group about the C–N bond is related to the disruption of favorable intramolecular interactions between the amine H-bond donor and imine N acceptor atoms. This barrier height is larger than the molecule energy difference between the two conformers of nitrofural, **3*α*** and **3*β***/**3*γ***. The values of the total molecule energy differences of both conformers suggest that a more stable molecular conformation is this one adapted by nitrofural in the forms **3*β*** and **3*γ*** (the difference between the energy of these forms and **3*α*** was 9.6 kcal/mol; value from the calculation done for optimized molecular geometry with the use of the M06-2X density functional). In the known polymorph **3*α***, where the nitrofural exists in another conformation, the hydrogen bond donors and acceptors point in two different directions, and this is the reason for the distinct hydrogen bond pattern. The intramolecular repulsions between the electronegative atoms with a lone pair of electrons can be responsible for the smaller stability of this conformer.

According to the conformational energy profile, furfural thio- and semicarbazones can adopt two energetically preferred equivalent conformations, in which the imine N atom and furan O atom can be syn- or anti-periplanar. These two conformations were observed in the studied compounds, but the syn-periplanar arrangement of heteroatoms was observed only in **3*α***. The analysis of structures in the Cambridge Structural Database (CSD) [25] shows that the syn-periplanar isomer is the predominant isomer for furfural thio- and semicarbazones. For thio- and semicarbazones of 2-thiophenecarboxaldehyde (thenaldehyde), the anti-periplanar isomer was not observed, neither for the studied compounds nor the CSD structures. According to the conformational energy profile, such conformation was less preferred in comparison to that of the syn-periplanar arrangement of N(imine) and S(thiophene) atoms. The conformational preferences of the studied compounds are similar to those exhibited by aldehyde itself. As in the studied compounds, the pure furfural exists as *cis* and *trans* conformers, whereas the *cis* form of pure thenaldehyde is favored. The character of electrostatic interactions between imine nitrogen and ring heteroatom is the major factor determining the more stable isomer. The stereoelectronic effects cause one conformation of thenaldehyde derived compounds (**2** and **4**) to be predominant as a result of the presence of a larger atom of sulfur (in comparison to the furfural oxygen atom) and subsequently a more favorable and rigid arrangement of a nitrogen one lone electron pair between two lone electron pairs of sulfur. The blockage of the nitrogen electron pair is smaller in the case of compounds possessing a furan ring as the distance between respective N and O atoms is smaller (d[N(imine)•••O(ring)] = 2.70 Å in **3*α***) than the distance between respective N and S atoms (d[N(imine)•••S(ring) = 3.07 and 3.02 Å in **2** and **4**, respectively). This is also one of important reasons that make both studied derivatives of thenaldehyde (**2** and **4**) isostructural.

### 2.3. Crystal Packing 

The Full Interaction Maps (FIMs) software was used to compare the existing non-covalent interactions formed by the studied compounds with the preferred intermolecular interactions occurring in similar compounds. The maps were generated using an uncharged NH donor, carbonyl, and oxygen acceptor probes. For compounds **1** and **2** possessing the thiosemicarbazone group, which showed a high propensity to form a thioamide dimer synthon, the analysis was expanded in the study of the thiocarbonyl contact group performed with the IsoStar Intermolecular Contact Database (IsoStar ICD), as FIMs software are unable to reproduce the respective maps. 

The thiosemicarbazones show a preference for the formation of homomeric dimers through both the primary (in 41%) and secondary (in 40%; observed also in **1** and **2**, Table S3) amine group with the thiocarbonyl S atom, whereas semicarbazones formed the amide(–C(=O)–NH–)···amide(–C(=O)–NH–) homosynthon more frequently (this synthon was observed in 49% of compounds; observed also in **4**, Table S3) [25]. The heteromeric dimer formed by thioamide(–C(=S)–NH_2_)···thioamide(–C(=S)–NH-) and amide(–C(=O)–NH_2_)···amide(–C(=O)–NH-) groups was rarely observed (only for 10% of crystal structures; observed also in **3*β***).

The analysis of the calculated interaction maps and contoured interaction–density plots (Figure S2) showed that the studied compounds formed interactions consistent with the interaction preferences. The lack of high-probability areas for the hydrogen bond acceptors and the clearly located areas for hydrogen bond donors near the thioamide group on FIMs (for **1** and **2**, Table S3) is the result of (a) the limitation in the choice of functional groups to be used as probes and (b) the high propensity of the thioamide group to form a ring dimer synthon via N–H···S hydrogen bonds. The geometry of the thioamide group intermolecular interactions for **1** and **2** (Table S3) is also typical, as shown in the contoured density surface plots obtained from the IsoStar ICD of intermolecular interactions. The distributions of thiocarbonyl acceptors and NH donors around the thiosemicarbazone group suggest that both the primary and secondary amine groups are involved in hydrogen bonding with the C=S group and the hydrogen atoms of the primary amine group are non-equivalent, with one of them forming hydrogen bonds more frequently. For **1** and **2**, the preferred thiocarbonyl group position near the primary amine is replaced by a nitro group. The molecules of **1** and **2** form only homomeric dimers through secondary amine and thiocarbonyl groups. For both polymorphic forms of **1·dmf** solvate, the dimer motif was not observed. The solvent carbonyl group position is typical for preferred oxygen atom location, but the thiocarbonyl acceptor forms interactions (of less favored geometry) with the primary amine group. In all cases, the nitro group forms intermolecular interactions of less common geometries. Typically, the nitro group forms interactions via both oxygen atoms with one donor placed in the area of O–N–O angle bisector.

The differences between nitrofural polymorphs (**3**) concern the NH_2_ donor. The **3*γ*** does not possess an acceptor near one of the hydrogen atoms of the amine group. The strong interaction map peaks corresponding to an acceptor near each of the hydrogen atoms of the amine group indicate the high propensity of this group to form interactions with noticeably preferred geometry. The presence of this unsatisfied hydrogen-bond donor can explain the reason for the lower possibility of this form being created. For **3*α***, the small donor hot spot near the amine group is occupied by a furan oxygen atom, which suggests that such a position of the acceptor near the amine group is statistically less probable. 

To verify the likelihood of the observed hydrogen bond formation for molecules of **1**–**4** and **1** solvate, the hydrogen bond propensities were calculated (Table S4). The NH_2_···O/S=C hydrogen bonds with the highest propensity values were not observed in the studied compounds (**1**, **2**, **4**). The NH_2_···O=C hydrogen bonds only existed in the crystal structures of two polymorphic forms of nitrofural (**3*α*** and **3*β***). The hydrogen bonds with high propensity existing in all four compounds involved the hydrazinic NH group and carbonyl/thiocarbonyl group. The observed interactions between the amide NH_2_ group and the nitro O atom also had high propensity, but the formation of analogous interactions between the hydrazinic NH group and the nitro O atom is highly improbable (taking into account the low value of propensity), and accordingly, they do not exist in compounds **1**–**4**. The observed NH_2_(amide)···N(imine) and NH_2_(amide)···O(furan) interactions in **3*α*** are very rare, and they are only low propensity interactions existing in the crystal structures of **1**–**4**. This might be a reason for the problems with the formation of **3*α*** during the synthesis and crystallization.

Both polymorphic forms of **1·dmf** solvate showed the most probable combination of hydrogen bonds. In contrast to **1**, the interactions between the amide NH_2_ group and the thiocarbonyl group (showing high propensity) are present in the **1·dmf** solvate. Moreover, the hydrazinic NH group forms more possible interactions with the solvent carbonyl group, which is an alternative to the thiocarbonyl acceptor of **1**.

**Figure 3:**
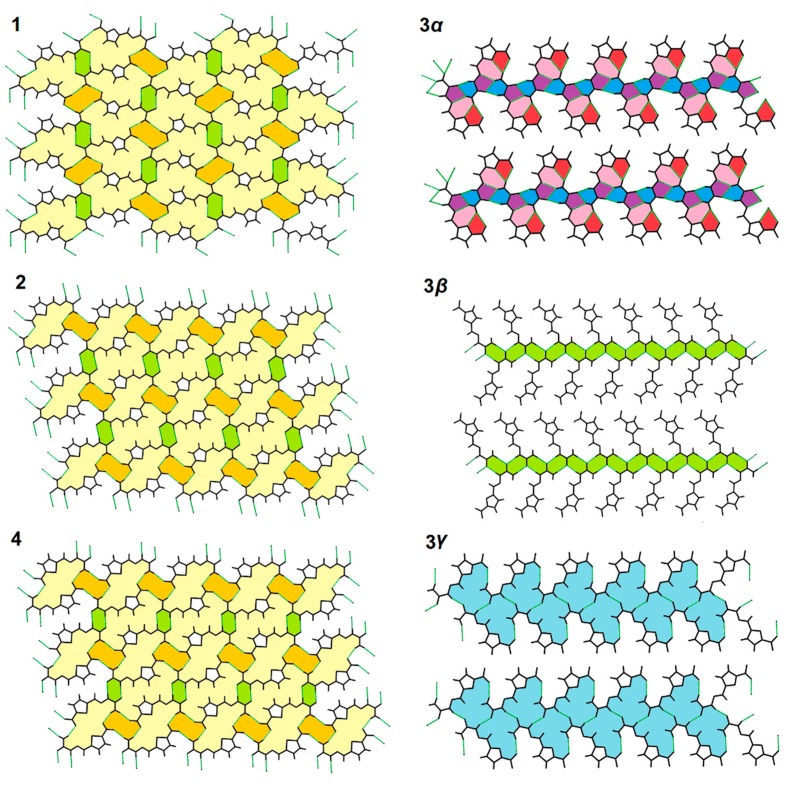
Tessellations in the studied compounds, created via hydrogen bonds assembling molecules in an appropriate crystallographic plane: (1 0 1) for **1** and **3*α***, (2 1 1) for **2** and **4**, (3 0 1) for **3*β***, (1 0 2) for **3*γ*** (for detailed description see text).

The analysis of tessellation confirmed the above findings. The hexagonal tiles (green, Figure 3) were created in compounds **1**, **2**, **3*β***, and **4**. It is a sole motif tile in a predominant plane in **3*β***, whereas in **1**, **2**, and **4** exist two additional motif tiles (yellow and orange, Figure 3). These tiles have identical shapes but different arrangements (i.e., in **1** they are oriented alternatively in each subsequent column, while in **2** and **4** they have the same alignment). The **3*γ*** also had only one motif tile in the predominant plane (similarly to **3*β***), but had a more irregular shape than the above-mentioned hexagon. **3*α*** possesses uncommon pentagonal and heptagonal tiling.

### 2.4. Polymorph Stability

Structures of the nitrofural (**3**) polymorphs possessed different packing than the **1·dmf** solvate polymorphs. In three polymorphic forms of **3**, hydrogen bonds play a substantial role in controlling the arrangement of the molecules in solid state (Table S3). The conventional N–H···O hydrogen bonds existed in all polymorphs, but formed different hydrogen bonding patterns. In general, the number of hydrogen bonds correlates with the crystal density and packing efficiency and these values are smaller for smaller numbers of hydrogen bonds. The values of the lattice energy (Table 2) of crystals **3*β*** and **3*γ*** were similar (with difference of 0.3 kcal/mol) and different from **3*α***, for which the strongest intermolecular interactions were observed, and the lattice energy was significantly larger in magnitude. Typically, larger lattice energy differences are observed for conformational polymorphs than for polymorphs possessing identical conformers [26]. The contribution from Coulombic-polarization energy to attractive forces was bigger for **3*α***, due to multiple hydrogen bonds, while the dispersion energy contribution was smaller for this form. The dispersion interactions had a relevant contribution to attractive interactions existing in the polymorphic forms of compound **3**, although, on the basis of geometrical parameters, classical *π*···*π* pile stacking interactions do not exist in these compounds (i.e., the interactions between *π* orbitals of the cyclic molecular moieties, in which the centroids of these moieties are separated at distance shorter than 4 Å) [27,28]. Only very weak interactions at larger Cg···Cg distances [29,30] were observed in the structures of compound 3. These interactions can be considered as formed between the bonding π orbitals of one ring donating electron density mainly to the antibonding π orbitals of the second ring (formal *π*···*π* interactions) and at a smaller part to the one-center Rydberg antibonding orbitals of the π-bonded atom of the second ring (formal co-participation of C−H···π and π···π interactions) [29]. The stability of the polymorphs of **3** cannot be determined on the basis of melting points because only two polymorphs can be currently produced, and both melt with decomposition.

Taking into account the conformational flexibility of nitrofural compounds and lattice energy values, three polymorphs represented the two different types of interplay between the molecular and crystal structure. Two forms (**3*β*** and **3*γ***) adopt lower energy conformation, which is more efficiently packed in the crystal lattice, but creates a limited number of classical hydrogen bonds (in comparison to **3*α***). **3*α*** exists as a higher energy conformer (with different positions of functional groups taking part in hydrogen bonds formation), which is less efficiently packed in the crystal lattice in comparison to **3*γ***, but its stabilization is possible due to intermolecular interactions that allows for stable crystal packing. The comparison of the energy differences between the two observed conformations of **3** (the geometrically optimized molecule of **3*α*** was 9.6 kcal/mol energetically less favored than **3*β*** and **3*γ***; Table 2) and the difference between the lattice energy of the polymorphic forms (**3*β*** net was 10.8 kcal/mol energetically less stable than the net of **3*α***, while respective value for **3*γ*** and **3*α*** was 10.5 kcal/mol; see E_latt_+E_M06-2X_ values in Table 2) proved that the stabilization energy of the lattice was close to the energy difference between the favored and unfavored conformation of **3** (at 0.9–1.2 kcal/mol). Analysis of the differences of the sum of lattice energy and Hartree–Fock energy with the inclusion of the Møller–Plesset correlation energy correction (calculated for atom positions taken from x-ray coordinates registered at room temperature after optimization of the hydrogen atoms positions; see E_latt_ + E_MP2_ values in Table 2) showed that the solid state forms **3*β*** and **3*γ*** were 3.6 and 4.0 kcal/mol, respectively, more stable than **3*α***. This can also explain the problems with the experimental reproduction of the form **3*α***, as the formation of **3*α*** in the solid state requires the adoption of energetically less favorable (in comparison to **3*β*** and **3*γ***) conformation during crystallization. Nevertheless, all these forms possessed similar total energy (including the influence of components originating from crystal lattice and molecular conformation).

For both polymorphic forms of **1·dmf** solvate, the calculated density, packing index, and lattice energy were similar. The differences in crystal packing motifs for these two polymorphs were mainly reflected in the energetic characteristics. In contrast to **1·dmf*β***, for **1·dmf*α***, whose molecules are stacked in piles along the *a* axis, the dispersive component is greater for the intermolecular interactions between the molecules of **1** and between the solvent molecules, at the expense of a smaller dispersive contribution related to the interactions between the molecules of **1** and molecules of the solvent. 

### 2.5. IR Spectra

The IR spectra of the studied compounds were generally similar due to their isomorphicity (Figure S3), but some clear shifts of bands were observed as a result of the different conformation of molecules and different intermolecular interactions (Table S5) [31]. All discussed spectra were registered for the synthesized compounds in transmission mode to the maximal decrease of noise (in previous works, the spectra were registered in reflectance mode). The formation of intramolecular hydrogen bonds by the NH_2_ and N(imine) acceptor further interacted with the thiophene S atom leads to a blue shift of *ν*_as_ NH_2_ vibrations present in the spectra of **2** and **4** (in comparison to other compounds, Table S5). A similar blue shift was observed for the *ν* CC_(ring)_ vibrations (which exists in **2** and **4** at about 1585 cm^−1^, together with *σ* NH_2_ vibrations) as a result of different electron withdrawing from the furan and thiophene rings by the NO_2_ substituent (the more electron rich thiophene ring allows for the redirection of more electron density to the NO_2_ group and stabilize the respective bonds restraining their vibrations). These effects caused a reverse (red) shift of the *ν* C=S vibration (weakening of the bond) in **2** in comparison to **1** and both of its solvates (**1·dmf**). Such a red shift (in **2** and **4**) was also clearly visible for the *ρ* NH_2_ vibrations due to the reasons given above. In the far IR spectra, the presence of the oscillations of hydrogen bonds formed by the NH_2_ group (NH_2_•••O_2_N and NH_2_•••O=C vibrations) caused an appearance of weak bands in the range of 359–376 cm^-1^. This proves that these bonds are the strongest ones in the studied compounds, and is subsequently in agreement with the relative strength of the respective hydrogen bondsdonors and acceptors. The NH•••O=C(dmf) vibrations can be clearly distinguished as they can appear only in forms of **1·dmf**. Respective bands were red shifted in comparison to the bands of hydrogen bonds formed by the NH_2_ donor as a result of weaker interactions created by the NH secondary amine donor. Further oscillators created by sets of hydrogen bonds were observed at smaller wavelengths. However, it is hard to unambiguously assign specific oscillators to exact bands due to the similar strength of other (than the above-mentioned) hydrogen bonds. The spectra of compound **3*α*** were not registered due to the impossibility of reproducing synthesis of this form and the far IR spectra of compounds **3*β*** and **3*γ*** had no clearly visible bands in this region.

## 3. Materials and Methods

### 3.1. Synthesis

Equimolar quantities of thiosemicarbazide (1 mmol) and the appropriate carbonyl compound (1 mmol of 5-nitro-2-furaldehyde and 5-nitro-2-thiophene, respectively) were dissolved in methanol (30 mL). The solution mixtures were heated with continuous stirring for 20 min at 65 °C. The slow evaporation of the solvent at room temperature gave the crystalline products: 5-nitro-2-furaldehyde thiosemicarbazone (**1**) and 5-nitro-2-thiophene thiosemicarbazone (**2**). 5-Nitro-2-furaldehyde semicarbazone (**3*β***) and 5-nitro-2-thiophene semicarbazone (**4**) were synthesized by the same procedure, only semicarbazide hydrochloride (1 mmnol) was used instead of thiosemicarbazide. Solid samples of the synthesized compounds (**1**–**4**) were dissolved in a pure solvent (MeOH, EtOH, i-PrOH, MeCN, or dmf) or solvent mixture (dmf + MeOH, dmf + EtOH, dmf + i-PrOH, dmf + MeCN, or MeOH + MeCN; 1 + 1 *v/v* in all cases) and heated for 10 minutes and then left for crystallization under ambient conditions (in covered, but not sealed, vessels to allow for the decelerated evaporation of solvents). Further structurally different crystalline products were obtained by the slow evaporation of solvents only for **1** and **3** (i.e., 5-nitro-2-furaldehyde thiosemicarbazone dimethylformamide dimethylformamide solvate (**1·dmfα**, by slow evaporation from dmf solution), 5-nitro-2-furaldehyde thiosemicarbazone dimethylformamide solvate (**1·dmf*β***, by slow evaporation from MeOH:dmf (1:1 *v/v*) solvent mixture), and 5-nitro-2-furaldehyde semicarbazone (**3*γ***, by slow evaporation from MeOH:MeCN (1:1 *v/v*) solvent mixture as well as from pure MeCN)). 

### 3.2. Crystal Structure Determination

The crystals of the studied compounds were mounted in turn on a Rigaku Synergy Dualflex automatic diffractometer equipped with a Pilatus 300K detector and used for data collection. X-ray intensity data were collected with mirror monochromated Mo*K_α_* (λ = 0.71073 Å) or Cu*K_α_* (λ = 1.54184 Å) radiation generated in a micro-focus sealed PhotonJet x-ray tube. The shutterless *ω* scan mode was used, and reflections were collected at temperature of 100.0(1) K. The unit cell parameters were determined from the strongest reflections. Details concerning the crystal data and refinement are given in Table 1 for the newly determined structures and Table S1 for the redetermined structures. Examination of the same reference reflections measured before and after measurement showed no loss of the intensity during measurements. Lorentz, polarization, and empirical absorption corrections (using spherical harmonics, implemented in SCALE3 ABSPACK scaling algorithm) were applied during the data reduction. The structure was solved by the dual-space algorithm. All the non-hydrogen atoms were refined anisotropically using the full-matrix, least-squares technique on *F*^2^. All hydrogen atoms were found from difference Fourier synthesis after four cycles of anisotropic refinement. Carbon bonded hydrogen atoms were refined as “riding” on the adjacent atom with geometric idealization after each cycle of refinement. Individual isotropic displacement factors of H atoms were set to be equal to 1.2 times the value of the equivalent displacement factors of the parent non-methyl carbon atoms, and 1.5 times that of the parent methyl carbon atoms. Nitrogen bonded hydrogen atom positions were freely refined (except one case described in the supplementary CIF file) and individual isotropic displacement factors were fixed to 1.2 of the value of equivalent displacement factors of the parent atoms. The methyl groups were allowed to rotate about their local three-fold axes. The SHELXT [32], SHELXL [33], and SHELXTL [34] programs were used for all calculations. Atomic scattering factors were taken from the International Tables for Crystallography [35]. Selected interatomic bond distances are listed in Table S2. 

CCDC 1976784-1976790 contain the supplementary crystallographic data for this paper. These data can be obtained free of charge via http://www.ccdc.cam.ac.uk/conts/retrieving.html (or from the CCDC, 12 Union Road, Cambridge CB2 1EZ, UK; Fax: +44-1223-336033; Email: deposit@ccdc.cam.ac.uk)

### 3.3. IR Spectrometry and Computational Methods

The IR spectra of the coordination compounds were recorded on a Jasco FT/IR 6200 spectrophotometer, in the form of KBr pellets for the spectral range 4000–400 cm^−1^ and in the form of CsI pellets for the spectral range of 400–200 cm^−1^.

The geometric parameters of the compounds were employed from the crystal structure data. The calculations of the total molecule energy of polymorphs of **3** and the conformational energy profile were performed in GAUSSIAN09 [36] using M06-2X density functional [37], the MP2 method [38], and the 6-31+g(d,p) basis set. Calculations using the MP2 and DFT methods showed the same energetic trend for each conformer, although the MP2 calculations predicted lower energy barriers to rotation. The experimentally obtained geometry was also used as input for the calculation of the lattice energy and its components (total lattice energy divided into individual coulombic, polarization, dispersion, and repulsion contributions to the interaction energies between each two-molecule system) using the Pixelc module of the Coulomb-London-Pauli (CLP) computer program package [39]. Before the calculations, the positions of the hydrogen atoms (determined form the x-ray diffraction experiments) were reset to provide the correct assignment of the requested parameters and values (e.g., atomic point charges). The required molecular electron densities were calculated using the MP2 [38] method with a 6-31G(d,p) basis set in the GAUSSIAN09 package [36] in the form of discrete values forming a three-dimensional grid (the cube). The net energies were calculated on the basis of numerical integration over the respective number of units of electron densities. The applied numerical integrals corresponded to the equations’ analytical form to provide parameter-free and accurate reproduction of wavefunction values. The delocalization of electrons (omitted in point-charge methods due to their nature) were assessed in terms of penetration energy to incorporate all parts of Coulombic energy into calculations. The polarization terms were computed on the basis of the linear dipole approach [39] and dispersion terms on the basis of London’s inverse sixth power calculation (comprising ionization potentials and polarizabilities) [40].

The Full Interaction Maps tool [41] and Polymorph Assessment tool [42] implemented in Mercury software [43] were used to evaluate the preferred interactions of molecules, based on pre-extracted IsoStar [44] interaction data from the CSD [25] and to ranking donors and acceptors based on propensity scores, respectively. 

## 4. Conclusions

The studied isomorphous molecules derived from 2-furaldehyde/2-thiophene and semi-/thiosemicarbazone exist in three cases in one polymorphic form (**1**, **2**, and **4**) and in one case in three polymorphic forms (**3**). The recrystallization of the studied compounds from different solvents gave solvates only in one case (of **1**) and with only one solvent (dmf). This, together with almost identical conformations of **1**, **1·dmfα**, **1·dmf*β***, **3*β***, and **3*γ*** implies that the presence of the thiocarbonyl donor and 2-thiophene ring is a necessary condition to create a solvate. On the other hand, the formation of two polymorphic forms of **1·dmf** solvate (possessing the same conformation of the constituent molecules and the same composition of crystal) proves that polymorphism in this case is caused solely by the intermolecular interactions existing in the crystallization environment (provided by OH groups of methanol molecules). 

The present study outlines that adopting a conformation of the lowest energy is not necessary to form a stable polymorphic form, as energy gained during the formation of the crystal net from molecules with higher energy can be greater than the differences between the adopted and the lowest energy confirmation. Additionally, the study proved that the presence of specific electronic restrains can be an important factor in diminishing the number of observed polymorphic forms. In the discussed case, one lone electron pair of nitrogen is located between two lone electron pairs of sulfur (compounds **2** and **4**). This reduces the flexibility of molecules and causes the presence of only one conformation for **2** and **4** (Figure 2). Subsequently, it led to the creation of only one polymorphic form (for **2** and **4**) under the studied conditions.

## Figures and Tables

**Table 1:** Crystal data and structure refinement details for the studied compounds.

Compound	1	1·dmf*α*	1·dmf*β*	2	4
CCDC number	1976784	1976786	1976789	1976785	1976790
Empirical formula	C_6_H_6_N_4_O_3_S	C_9_H_13_N_5_O_4_S	C_9_H_13_N_5_O_4_S	C_6_H_6_N_4_O_2_S_2_	C_6_H_6_N_4_O_3_S
Formula weight	214.21	287.30	287.30	230.27	214.21
Crystal system	Monoclinic	Monoclinic	Monoclinic	Triclinic	Triclinic
Space group	*P*2_1_/*n* (No. 14)	*P*2_1_/*n* (No. 14)	*Cc* (No. 9)	*P*-1 (No. 2)	*P*-1 (No. 2)
Temperature (K)	100.0(1)	100.0(1)	100.0(1)	100.0(1)	100.0(1)
Wavelength (Å)	0.71073 *λ*(Mo*Kα*)	1.54184 *λ*(Cu*Kα*)	0.71073 *λ*(Mo*Kα*)	0.71073 *λ*(Mo*Kα*)	0.71073 *λ*(Mo*Kα*)
Unit cell dimensions					
a (Å)	12.262(3)	4.7031(1)	5.9513(4)	4.5364(1)	4.5040(2)
b (Å)	12.4011(14)	24.9324(5)	19.6479(15)	8.7380(2)	8.5940(3)
c (Å)	12.670(3)	11.1468(2)	11.4607(7)	12.2261(3)	11.7247(4)
α (°)	90.00	90.00	90.00	76.291(2)	71.920(3)
β (°)	113.84(3)	98.552(1)	104.431(5)	87.644(2)	86.928(3)
γ (°)	90.00	90.00	90.00	79.401(2)	77.680(3)
Volume (Å^3^)	1762.3(6)	1292.54(3)	1297.82(16)	462.791(19)	421.45(3)
Z	8	4	4	2	2
Calculated density (Mg/m^3^)	1.615	1.476	1.470	1.652	1.688
Absorption coefficient (mm^−1^)	0.354	2.435	0.269	0.553	0.370
*F(000)*	880	600	600	236	220
Crystal size (mm)	0.098 0.090 0.007	0.173 0.131 0.118	0.121 0.084 0.024	0.107 0.098 0.091	0.098 0.093 0.025
*θ* Range for data collection (°)	3.416 to 25.049	4.386 to 78.482	3.671 to 32.078	3.430 to 31.793	3.563 to 31.940
Index ranges	−14 ≤ h ≤ 14, −14 ≤ k ≤ 14, −15 ≤ l ≤ 14	−5 ≤ h ≤ 5, −31 ≤ k ≤ 31, −14 ≤ l ≤ 13	−8 ≤ h ≤ 8, −28 ≤ k ≤ 28, −16 ≤ l ≤ 14	−6 ≤ h ≤6, −12 ≤ k ≤ 12, −17 ≤ l ≤ 17	−6 ≤ h ≤ 6, −12 ≤ k ≤ 12, −17 ≤ l ≤ 17
Reflections collected/unique	14539/3111	25641/2646	12573/3526	12959/2743	12046/2544
R*_int_*	0.0647	0.0350	0.0464	0.0278	0.0338
Completeness (%) to *θ* = 25°(Mo) and *θ* = 67°(Cu)	99.8	100.0	99.8	99.8	99.8
Min. and max. transmission	0.48113 and 1.00000	0.63893 and 1.00000	0.86402 and 1.00000	0.94704 and 1.00000	0.96223 and 1.00000
Data/restraints/parameters	3111/1/271	2646/0/183	3526/2/183	2743/0/136	2544/0/136
Goodness-of-fit on *F*^2^	1.044	1.043	1.084	1.078	1.050
Final *R* indices [*I* > 2σ(*I*)]	*R*1 = 0.0632, *wR*2 = 0.1595	*R*1 = 0.0271, *wR*2 = 0.0698	*R*1 = 0.0357, *wR*2 = 0.0951	*R*1 = 0.0249, *wR*2 = 0.0658	*R*1 = 0.0282, *wR*2 = 0.0725
R indices (all data)	*R*1 = 0.0834, *wR*2 = 0.1705	*R*1 = 0.0289, *wR*2 = 0.0708	*R*1 = 0.0366, *wR*2 = 0.0957	*R*1 = 0.0290, *wR*2 = 0.0674	*R*1 = 0.0337, *wR*2 = 0.0745
Largest diff. peak and hole (e•Å^−3^)	0.995 and −0.352	0.414 and −0.205	0.582 and −0.546	0.425 and −0.233	0.428 and −0.229

**Table 2:** The packing index and density values derived from structural data. E_latt_ is the calculated total lattice energy (as −ΔH of sublimation) and its components. E_MP2_ is the Hartree–Fock energy with the inclusion of the Møller–Plesset correlation energy correction calculated for the atom positions taken from x-ray coordinates registered at room temperature after optimization of the hydrogen atoms positions. E_M06-2X_ is the energy calculated with the M06-2X density functional with optimization of whole molecule geometry. Energy values are in kJ/mol.

Compound	Packing Index (%)	Density (g/cm^3^)	E_latt_	E_Coul_	E_pol_	E_disp_	E_rep_	E_MP2_	E_M06-2X_
								E_latt_+E_MP2_	E_latt_+E_M06-2X_
**3*α***	69.9	1.571	−178.6	−148.4	−60.3	−117.3	147.3	−1968944	−1973692
								−1969123	−1973871
**3*β***	68.8	1.547	−133.4	−107.1	−38.2	−109.9	126.6	−1968999	−1973732
								−1969132	−1973865
**3*γ***	71.2	1.592	−134.7	−99.7	−33.9	−116.1	114.9	−1969005	−1973732
								−1969140	−1973867
**1**	—	—	—	—	—	—	—	−2815948	−2821619
								—	—
**1·dmf*α***	71.2	1.476	−107.6	−81.3	−33.3	−100.2	107.2	−3466466	—
								−3466574	—
**1·dmf*β***	70.6	1.470	−109.1	−84.0	−33.5	−101.2	111.8	−3466466	—
								−3466575	—

## Supplementary Materials

The supplementary materials are available online.
