# Supplementary material for: Interplay between Polymorphism and Isostructurality in the 2-Fur- and 2-Thenaldehyde Semi- and Thiosemicarbazones
Source: Molecules. 2020 Feb 23;25(4):993. doi: 10.3390/molecules25040993 (PMC7070665; doi:10.3390/molecules25040993)
Supplement: Supplementary file 1 [file molecules-25-00993-s001.pdf]

molecules

Supplementary Materials

Interplay between polymorphism and isostructurality in the 2-fur- and 2-thenaldehyde semi- and thiosemicarbazones

Marcin Swiatkowski, Agata Trzesowska-Kruszynska, Agnieszka Danielewicz, Paulina Sobczak and Rafal Kruszynski

Table S1. Crystal data and structure refinement details for the studied compounds.

Compound	**3*β***	**3*γ***
CCDC number	1976788	1976787
Empirical formula	$C_6H_6N_4O_4$	$C_6H_6N_4O_4$
Formula weight	198.15	198.15
Crystal system	Monoclinic	Monoclinic
Space group	$P2_1$ (No. 4)	$P2_1/c$ (No. 14)
Temperature (K)	100.0(1)	100.0(1)
Wavelength (Å)	1.54184<br>λ(Cu*Kα*)	1.54184<br>λ(Cu*Kα*)
Unit cell dimensions		
a (Å)	4.2045(9)	13.4461(11)
b (Å)	6.8953(12)	7.8484(10)
c (Å)	14.200(2)	7.8332(11)
α (°)	90.00	90.00
β (°)	92.683(6)	103.398(10)
γ (°)	90.00	90.00
Volume (Å^3)	411.23(13)	804.14(17)
Z	2	4
Calculated density (Mg/m^3)	1.600	1.637
Absorption coefficient (mm^{-1})	1.192	1.219
F(000)	204	408
Crystal size (mm)	0.092<br>0.090<br>0.042	0.107<br>0.068<br>0.063
θ Range for data collection (°)	3.115 to 68.423	3.379 to 72.240
Index ranges	$-4 \le h \le 5$,<br>$-8 \le k \le 8$,<br>$-16 \le l \le 16$	$-16 \le h \le 16$,<br>$-9 \le k \le 9$,<br>$-9 \le l \le 9$
Reflections collected / unique	4420 / 1434	8744 / 1592
R_{int}	0.0226	0.0177
Completeness to θ = 67° (%)	98.1	99.8
Min. and max. transmission	0.63647 and 1.00000	0.65240 and 1.00000
Data / restraints / parameters	1434 / 1 / 136	1592 / 0 / 136
Goodness-of-fit on F^2	1.087	1.106
Final *R* indices [I>2σ(I)]	$R1$ = 0.0302,<br>$wR2$ = 0.0759	$R1$ = 0.0301,<br>$wR2$ = 0.0812
R indices (all data)	$R1$ = 0.0302,<br>$wR2$ = 0.0760	$R1$ = 0.0302,<br>$wR2$ = 0.0813
Largest diff. peak and hole (e•Å^{-3})	0.252 and -0.410	0.231 and -0.304

Table S2. Structural data of the studied compounds (Å, °).

		1		1·dmf*α*	1·dmf*β*	2	3*β*	3*γ*	4
i—j	m—n	d_{ij}	d_{mn}	d_{ij}	d_{ij}	d_{ij}	d_{ij}	d_{ij}	d_{ij}
C1—O1/S1	C11—O11	1.376(4)	1.374(4)	1.3729(15)	1.377(3)	1.7313(11)	1.367(2)	1.3744(12)	1.7261(11)
C4—O1/S1	C14—O11	1.371(4)	1.369(4)	1.3559(15)	1.358(3)	1.7260(11)	1.3575(19)	1.3528(12)	1.7232(11)
C1—C2	C11—C12	1.365(5)	1.375(5)	1.3677(18)	1.375(3)	1.3840(14)	1.368(3)	1.3706(14)	1.3855(14)
C2—C3	C12—C13	1.420(5)	1.424(5)	1.4128(18)	1.419(3)	1.4092(15)	1.405(3)	1.4154(15)	1.4149(14)
C3—C4	C13—C14	1.363(5)	1.356(5)	1.3530(19)	1.367(3)	1.3688(15)	1.348(3)	1.3564(15)	1.3683(15)
C4—N4	C14—N14	1.414(5)	1.423(5)	1.4172(17)	1.423(3)	1.4272(14)	1.410(3)	1.4161(13)	1.4301(14)
N4—O2	N14—O12	1.238(4)	1.240(4)	1.2351(16)	1.241(3)	1.2401(13)	1.227(2)	1.2324(12)	1.2384(13)
N4—O3	N14—O13	1.244(4)	1.241(4)	1.2328(16)	1.234(3)	1.2368(12)	1.237(2)	1.2346(12)	1.2360(12)
C1—C5	C11—C15	1.451(5)	1.443(5)	1.4414(17)	1.447(3)	1.4463(15)	1.448(2)	1.4417(15)	1.4505(14)
C5—N1	C15—N11	1.294(5)	1.301(5)	1.2851(17)	1.289(3)	1.2888(14)	1.275(2)	1.2875(14)	1.2919(14)
N1—N2	N11—N12	1.377(4)	1.374(4)	1.3600(14)	1.363(3)	1.3687(13)	1.360(2)	1.3584(12)	1.3664(12)
N2—C6	N12—C16	1.367(5)	1.368(5)	1.3605(16)	1.369(3)	1.3565(13)	1.361(3)	1.3704(14)	1.3801(13)
C6—S2/O4	C16—S12	1.699(3)	1.697(4)	1.6909(13)	1.693(2)	1.6953(11)	1.245(2)	1.2386(13)	1.2501(13)
C6—N3	C16—N13	1.330(5)	1.332(5)	1.3224(16)	1.330(3)	1.3280(14)	1.327(3)	1.3364(14)	1.3356(14)
i—j—k	m—n—o	α_{ijk}	α_{mno}	α_{ijk}	α_{ijk}	α_{ijk}	α_{ijk}	α_{ijk}	α_{ijk}
C1—C2—C3	C11—C12—C13	107.4(3)	106.5(3)	106.61(11)	106.4(2)	113.13(10)	107.0(2)	106.51(9)	113.11(10)
C2—C3—C4	C12—C13—C14	105.0(3)	105.8(3)	105.36(12)	105.2(2)	110.41(10)	105.19(17)	105.32(9)	109.94(9)
C3—C4—O1/S1	C13—C14—O11	112.1(3)	111.9(3)	112.66(11)	112.67(19)	114.77(8)	112.82(18)	112.69(9)	115.14(8)
C4—O1/S1—C1	C14—O11—C11	105.1(3)	105.4(3)	104.82(10)	104.82(17)	89.33(5)	104.71(15)	104.98(8)	89.36(5)
O1/S1—C1—C2	O11—C11—C12	110.3(3)	110.4(3)	110.54(11)	110.87(19)	112.35(8)	110.30(16)	110.50(9)	112.43(8)
N4—C4—O1/S1	N14—C14—O11	117.0(3)	116.8(3)	115.75(11)	116.79(19)	119.87(8)	116.73(17)	116.31(9)	118.69(8)
N4—C4—C3	N14—C14—C13	130.8(3)	131.2(3)	131.58(12)	130.4(2)	125.35(10)	130.40(16)	130.99(10)	126.16(10)
C4—N4—O2	C14—N14—O12	118.1(3)	118.0(3)	118.39(11)	118.4(2)	117.25(9)	118.73(15)	119.20(9)	117.26(9)
C4—N4—O3	C14—N14—O13	118.0(3)	118.5(3)	116.96(12)	116.7(2)	118.92(9)	116.45(16)	116.45(9)	119.08(9)
O2—N4—O3	O12—N14—O13	123.8(3)	123.5(3)	124.65(12)	124.9(2)	123.82(10)	124.82(17)	124.35(9)	123.65(10)
O1/S1—C1—C5	O11—C11—C15	113.9(3)	114.5(3)	115.82(11)	115.65(19)	122.94(8)	114.98(15)	114.55(9)	121.64(8)
C2—C1—C5	C12—C11—C15	135.7(3)	135.0(3)	133.64(12)	133.5(2)	124.71(10)	134.72(18)	134.89(10)	125.91(10)
C1—C5—N1	C11—C15—N11	119.3(3)	119.7(3)	117.07(11)	117.1(2)	121.13(10)	118.22(16)	119.02(10)	119.85(10)
C5—N1—N2	C15—N11—N12	115.3(3)	115.5(3)	116.75(11)	116.68(19)	114.80(9)	116.24(15)	115.59(9)	115.64(9)
N1—N2—C6	N11—N12—C16	120.5(3)	120.6(3)	118.90(10)	118.33(18)	120.46(9)	119.99(16)	120.00(9)	119.71(9)

N2—C6—N3	N12—C16—N13	117.1(3)	117.5(3)	117.21(11)	117.0(2)	117.50(10)	117.30(15)	116.95(9)	116.93(9)
N2—C6—S2/O4	N12—C16—S12	118.7(3)	119.1(3)	118.82(9)	119.49(16)	118.55(8)	118.48(18)	118.70(10)	118.67(10)
N3—C6—S2/O4	N13—C16—S12	124.2(3)	123.4(3)	123.97(10)	123.50(16)	123.95(8)	124.21(18)	124.35(10)	124.38(10)
i—j—k—l	m—n—o—p	τ_{ijkl}	τ_{mnop}	τ_{ijkl}	τ_{ijkl}	τ_{ijkl}	τ_{ijkl}	τ_{ijkl}	τ_{ijkl}
O1/S1—C1—C5—N1	O11—C11—C15—N11	-176.6(3)	178.6(3)	-178.58(11)	-177.5(2)	-2.44(15)	174.53(16)	-179.41(9)	3.64(14)
N1—N2—C6—N3	N11—N12—C16—N13	2.3(5)	-2.3(5)	3.60(17)	2.8(3)	-2.44(15)	2.7(3)	4.59(14)	3.79(15)

Table S3. Hydrogen bonds and S/O•••π interactions in the studied compounds [Å, °]. Each ring is indicated by one atom, which belongs to this ring (Cg indicates respective ring centroid).

D—H•••A	d(D—H)	d(H•••A)	d(D•••A)	<(DHA)
		**1**		
N2—H2N•••S12^{i}	0.97	2.37	3.330(3)	170
N3—H3A•••O2^{ii}	0.87	2.32	3.095(5)	149
N3—H3B•••O13^{iii}	0.72	2.47	3.184(5)	176
N12—H12N•••S2^{iv}	0.88	2.51	3.342(4)	159
N13—H13A•••O3^{v}	0.90	2.31	3.206(4)	172
N13—H13B•••O12^{vi}	0.88	2.17	3.044(5)	170
C2—H2•••O2^{ii}	0.95	2.56	3.435(5)	153
C3—H3•••S12^{v}	0.95	2.74	3.689(4)	174
C5—H5•••S12^{i}	1.06	2.76	3.705(4)	149
C12—H12•••O12^{vi}	0.95	2.54	3.409(5)	152
C13—H13•••S2^{iii}	0.95	2.75	3.688(4)	169
C15—H15•••S2^{iv}	0.88	2.94	3.713(4)	148
		**1·dmf*α***		
N2—H2N•••O11^{vii}	0.833	2.170	2.9576(14)	157.7
N3—H3A•••N1	0.859	2.293	2.6237(15)	103.0
N3—H3A•••S2^{viii}	0.859	2.622	3.3732(12)	146.6
N3—H3B•••O11	0.846	2.086	2.9281(15)	173.7
C2—H2•••S2^{viii}	0.95	2.99	3.9156(17)	165
C3—H3•••O3^{ix}	0.95	2.76	3.3997(18)	125
C5—H5•••O11^{vii}	0.95	2.54	3.3191(15)	139
C11—H11•••S2	0.95	2.88	3.6924(17)	144
C12—H12A•••S2^{x}	0.98	3.11	3.9051(17)	139
C12—H12B•••O2^{xi}	0.98	2.79	3.4154(18)	122
C12—H12C•••O3^{xii}	0.98	2.70	3.8569(18)	151
C13—H13A•••O11	0.98	2.44	2.8419(18)	104
C13—H13A•••O1^{iv}	0.98	2.65	3.2912(18)	123
C13—H13A•••O2^{iv}	0.98	2.89	3.5694(18)	127
C13—H13B•••O2^{xii}	0.98	2.54	3.3469(18)	139
		**1·dmf*β***		
N2—H2N•••O11^{x}	0.89	2.07	2.933(3)	165
N3—H3A•••N1	0.75	2.24	2.621(3)	112
N3—H3A•••S2^{viii}	0.75	2.70	3.288(2)	136
N3—H3B•••O11	0.89	2.03	2.913(3)	171
C2—H2•••S2^{viii}	0.95	3.14	4.080(3)	168
C3—H3•••O2^{xiii}	0.95	2.81	3.676(3)	156
C5—H5C•••O11^{x}	0.95	2.65	3.409(3)	137
C11—H11•••S2	0.95	2.90	3.741(3)	148
C12—H12B•••O1^{xiv}	0.98	2.60	3.449(3)	145
C12—H12C•••S2^{x}	0.98	3.05	3.772(3)	132
C12—H12C•••N3^{x}	0.98	2.87	3.490(3)	122
C13—H13A•••S2^{xv}	0.98	3.08	4.018(3)	161
C13—H13B•••O3^{xvi}	0.98	2.61	3.556(3)	161
C13—H13C•••O2^{iv}	0.98	2.71	3.564(3)	146
		**2**		
N2—H2N•••S2^{xvii}	0.834	2.561	3.3758(10)	165.7
N3—H3A•••N1	0.888	2.332	2.6640(14)	102.2
N3—H3A•••O2^{xviii}	0.888	2.138	2.9917(13)	161.0

N3—H3B•••O3^{xix}	0.850	2.511	3.3370(13)	164.1
C3—H3•••S2^{xx}	0.93	2.75	3.6006(13)	153
C5—H5•••S2^{xvii}	0.93	3.04	3.7852(13)	139
C5—H5•••S2^{xxi}	0.93	3.18	3.7391(13)	120
		**3*β***		
N2—H2N•••O4^{xxii}	0.87	1.94	2.787(2)	166
N3—H3A•••O2^{xxiii}	0.87	2.39	3.189(2)	153
N3—H3A•••N1	0.87	2.33	2.650(3)	102
N3—H3B•••O4 ^{xxiv}	0.86	2.04	2.883(2)	166
C2—H2•••O2^{xxiii}	0.95	2.86	3.731(3)	152
C3—H3•••O3^{xxv}	0.95	2.41	3.318(3)	161
		**3*γ***		
N2—H2N•••O4^{xxvi}	0.858	2.003	2.8197(13)	158.6
N3—H3A•••O2^{xxvii}	0.893	2.299	3.1590(14)	161.6
N3—H3A•••N1	0.893	2.298	2.6570(14)	103.9
N3—H3B•••O4^{xxviii}	0.868	2.830	3.4074(15)	125.3
C2—H2•••O2^{xxvii}	0.95	2.53	3.4313(14)	158
C3—H3•••O3^{xxix}	0.95	2.35	3.2243(14)	153
C5—H5•••O4^{xxvi}	0.95	2.42	3.2068(14)	139
		**4**		
N2—H2N•••O4^{xxx}	0.83	2.04	2.8662(1)	172.5
N3—H3A•••N1	0.98	2.28	2.6640(1)	102.3
N3—H3A•••O2^{xxxi}	0.98	2.12	3.0380(1)	154.3
N3—H3B•••O3^{xix}	0.97	2.12	3.0748(1)	170.3
C3—H3•••O4^{xx}	0.95	2.37	3.2818(1)	161.7
C5—H5•••O4^{xxx}	0.95	2.93	3.6211(1)	130.3

Y—X•••R(J)	d(X•••Cg)	d(Y•••Cg)	<(YXCg)
	**1**		
C6—S2•••R(O1)^{iii}	3.587(2)	3.732(4)	81.38(13)
C16—S12•••R(O11)^{viii}	3.741(2)	3.393(4)	65.01(13)
	**1·dmf*α***		
N4—O2•••R(O1)^{xxxii}	3.6171(11)	3.3686(11)	68.59(6)
N4—O3•••R(O1)^{xxxii}	3.2757(11)	3.3686(11)	83.58(7)
	**2**		
N4—O3•••R(S1)^{xxxii}	3.4588(10)	3.8053(11)	96.63(6)
	**3*β***		
N4—O3•••R(O1)^{xxxii}	3.2058(19)	3.3969(19)	88.07(10)
	**4**		
N4—O3•••R(S1)^{xxxii}	3.7216(10)	4.0332(11)	95.54(7)

Symmetry transformations used to generate equivalent atoms: (i) x+1.5, -y+0.5, z+0.5; (ii) -x+1.5, y+0.5, -z+0.5; (iii) -x+2, -y+1, z+1; (iv) x+1.5, -y+0.5, z-0.5; (v) -x, -y+1, -z; (vi) -x+0.5, y+0.5, -z+0.5; (vii) x-1.5, -y+0.5, z+0.5; (viii) x-0.5, -y+0.5, z-0.5; (ix) -x-1, -y+1, -z; (x) x+1, y, z; (xi) x+1.5, y-0.5, -z+0.5; (xii) -x+0.5, y-0.5, -z+0.5; (xiii) x, -y, z-0.5; (xiv) x+1.5, y+0.5, z; (xv) x+0.5, -y+0.5, z-0.5; (xvi) x+2.5, y+0.5, z; (xvii) –x+3, -y+1, -z; (xviii) –x+1, -y+1, -z+1; (xix) x+2, y-1, z; (xx) x-2, y+1, z; (xxi) –x+2, -y+1, -z; (xxii) -x+2, y-0.5, -z; (xxiii) x+1, y+1, z; (xxiv) -x+2, y+0.5, -z; (xxv) -x-1, y+0.5, -z+1; (xxvi) -x+1, y+0.5, -z+1.5; (xxvii) x, y-1, z; (xxviii) -x+1, -y, -z+1; (xxix) -x+2, y-0.5, -z+0.5; (xxx) -x+3, -y+1, -z; (xxxi) -x+1, -y+1, -z+1 (xxxii) x-1, y, z.

Table S4. Hydrogen bond propensity values for donor–acceptor pairs observed in the studied compounds.

Compound	Donor-acceptor pair	Propensity
**1**	NH···S=C	0.63
	NH_2···O(nitro)	0.40
**1·dmf*α***	NH_2···O=C(dmf)	0.90
**1·dmf*β***	NH_2···S=C	0.79
	NH···O=C(dmf)	0.75
**2**	NH···S=C	0.60
	NH_2···O(nitro)	0.52
**3*α***	NH_2···O=C	0.83
	NH···O=C	0.63
	NH_2···O(nitro)	0.55
	NH_2···N(imine)	0.18
	NH_2··· O(furan)	0.08
**3*β***	NH_2···O=C	0.83
	NH···O=C	0.63
	NH_2···O(nitro)	0.55
**3*γ***	NH···O=C	0.63
	NH_2···O(nitro)	0.55
**4**	NH_2···O(nitro)	0.62
	NH···O=C	0.59

Table S5. Vibrational frequencies and their assignments for the studied compounds. The data for **3** represents the calculated spectrum.

**1**	**1·dmf**α	**1·dmf**β	**2**	**3 [44]**	**3**β	**3**γ	**4**	**Assignment**
						3510 m		ν NH_2 (not involved in H-bonds)
3463 m	3464 w	3464 w	3473 m	3464	3461 m	3461 w	3484 m	ν_{as} NH_2
						3431 w		ν_{as} NH_2
3311 m	3311 w	3312 w	3315 m	3349	3355 w	3389	3370 m	ν_s NH_2
					3290 w			ν NH
3181 vw	3183 vw	3182 vw	3162 w	3240	3238 w	3263 w	3205 vw	ν NH
3117 w	3117 vw	3114 vw			3155 w	3151 w	3135 w	ν $CH_{(ring)}$
3087 vw	3087 w	3088 w	3069 w	3138	3135 w	3135 w	3065 m	ν $CH_{(ring)}$
3057 vw	3057 vw	3058 vw	3003 w		3123 w	3117 w	2991 w	ν $CH_{(ring)}$
2977 w	2975 vw	2978 vw	2960 vw	2957	2903 w	2955 w	2926 w	ν $CH_{(imine)}$, ν_{as} $CH_{3(dmf)}$
	2918 vw							ν_s $CH_{3(dmf)}$
	2850 w							ν $CH_{(dmf)}$
				1728	1715 s	1711 s	1679 s	ν C=O
					1682 w	1686 w		ν C=N
	1640 s	1640 s						ν $C{=}O_{(dmf)}$
1602 s	1603 s	1603 s	1608 s		1596 w	1596 w		ν C=N
1593 s	1593 w	1593 w	1585 w	1587	1582 s	1580 m	1584 s	σ NH_2, ν C=N,
1570 m	1570 w	1570 w		1561	1565 w	1566 s		ν $CC_{(ring)}$
1538 s	1538 s	1539 s	1543 s	1536	1537 w	1543 w	1538 w	ν_{as} NO_2
1506 s	1505 m	1507 m	1525 s	1505	1507 s	1499 s	1510 m	δ_{in} NH, ν $CN_{(dmf)}$
1480 s	1481 m	1481 m	1486 s					ν C=S, δ_{as} $CH_{3(dmf)}$
			1473 s	1472	1465 s	1470 s	1490 s	ν $CC_{(ring)}$, δ_{in} NH
1453 s	1454 s	1455 s	1434 s			1422 s	1431 s	δ_{in} NH
1394 s	1394 s	1394 s	1384 m	1396	1390 m	1390 m		ν $CC_{(ring)}$, δ_{in} $CH_{(imine)}$, δ_s $CH_{3(dmf)}$
			1368 m					ν $CC_{(ring)}$, δ_{in} $CH_{(ring)}$
1355 s	1357 s	1357 s	1336 s	1345	1349 s	1352 s	1334 s	ν_s NO_2
					1327 s	1332 s	1308 s	ν_{as} $NCN_{(amide)}$
				1319	1313 m	1315 m		δ_{in} $CH_{(imine)}$
1274 m	1274 w	1275 w	1266 m					ν C=S
1258 m	1258 m	1259 m	1233 m	1255	1252 s	1256 s	1223 s	ρ NH_2, ν $CN(NH_2)$, ν_{as} $C_2N_{(dmf)}$

**1**	**1·dmfα**	**1·dmfβ**	**2**	**3 [44]**	**3β**	**3γ**	**4**	**Assignment**
1190 m	1191 m	1191 m		1204	1200 s	1201 s		ν CC$_{(ring)}$, δ_{in} CH$_{(ring)}$
1105 m	1108 m	1108 m	1102 m	1153	1149 m	1149 m	1159 m	ν NN, ρ CH$_{3(dmf)}$
1058 m	1059 w	1059 w				1081 m		ν CC$_{(ring)}$, δ_{in} CH$_{(ring)}$, ρ CH$_{3(dmf)}$
1029 s	1030 m	1030 m	1043 m	1026	1020 s	1025 s	1052 m	ρ NH$_2$, ν CN(NH$_2$)
976 s	976 w	976 w			969 s	970 s	997 w	δ_{in} N-N=C, ν CO$_{(ring)}$
971 s	971 w	971 w		971				δ_{in} N-N=C, ν CO$_{(ring)}$
---		---	922 w			920 m		ω CH$_{(ring)}$
909 s	908 w	909 w	911 m	905	904 m	911 m	909 m	ω CH$_{(ring)}$, δ_{out} CH$_{(imine)}$
845 s	846 w	845 w	841 s					δ_{out} NH
829 s	828 w	829 w	815 s		820 m	829 m		δ_{out} NH
811 m	811 w	811 w	806 s	813	807 m	810 m	807 m	σ NO$_2$
777 s	777 w	778 w		784	786 m	785 m		ω CH$_{(ring)}$
			766 w				767 m	ν CS$_{(ring)}$
				764	760 w	759w		δ_{in} N=C-C
736 m	736 w	737 w	733 s	741	737 m	736 m	732 m	δ_{out} NH
705 w	703 vw	703 w	701 w	724			711 m	ω NO$_2$
			680 w				681 w	δ_{out} CH$_{(ring)}$
				665	651 w	657 w	640 w	δ_{in} C=O
655 m	654 w	654 w	635 m	656			620 w	T ring, δ_{in} C=O$_{(dmf)}$
603 w	603 vw	603 vw		595				T ring
569 m	570 w	570 w	579 w	561	594 m	573 m	577 m	ρ NO$_2$
	546 vw	546 vw	564 w					T ring
526 m	525 w	527 vw	530 m		550 m	550 m		ω NH$_2$
480 m	480 w	481 w	492 m	473	471 m	475 m	497 s	δ_{out} NH
	452 w	454 w	457 w					T ring
		440 vw			430 w	425 w		δ_{in} N=C-N
428 s	429 w	429 w	411 m		406 vw	413 vw	432 m	δ_{in} N-C-N
	409 w	409 w						σ C$_2$N$_{(dmf)}$
373 vw			376w				359 vw	NH$_2$•••O$_2$N
	375 w	375 w						NH$_2$•••O=C (dmf)

**1**	**1·dmf*α***	**1·dmf*β***	**2**	**3 [44]**	**3*β***	**3*γ***	**4**	**Assignment**
	328 vw	329 vw						NH•••O=C (dmf)
	302 vw	302 vw					292 w	
282 vw	281 w	281 vw	278 vw				283 w	
274 vw	266 vw	266 vw	270 vw					
247 vw	246 w	247 vw	256 vw				250 w	

Vibrations symbols: vw – very weak, w – weak, m – medium, s – strong, ν – stretching, δ – bending, σ – scissoring, ρ – rocking, τ – twisting, ω – wagging, T – torsional, s – symmetric, as – asymmetric, in – in-plane, out – out-of-plane.

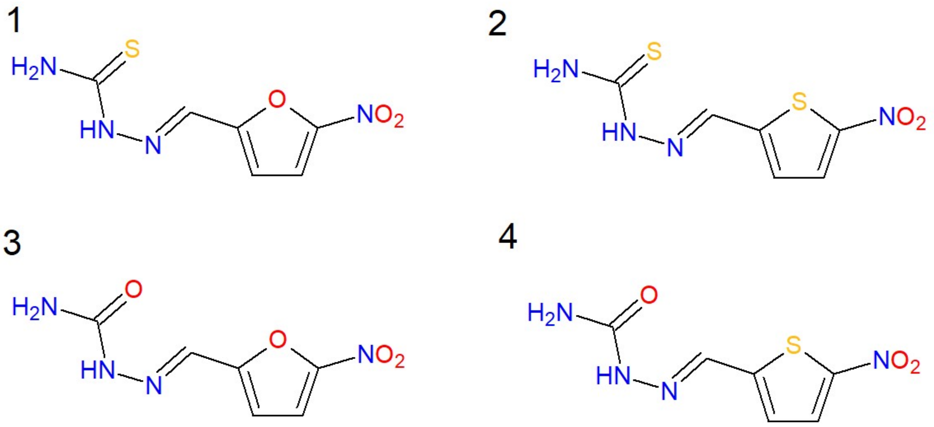

Scheme S1. The chemical formulas of the studied compounds.

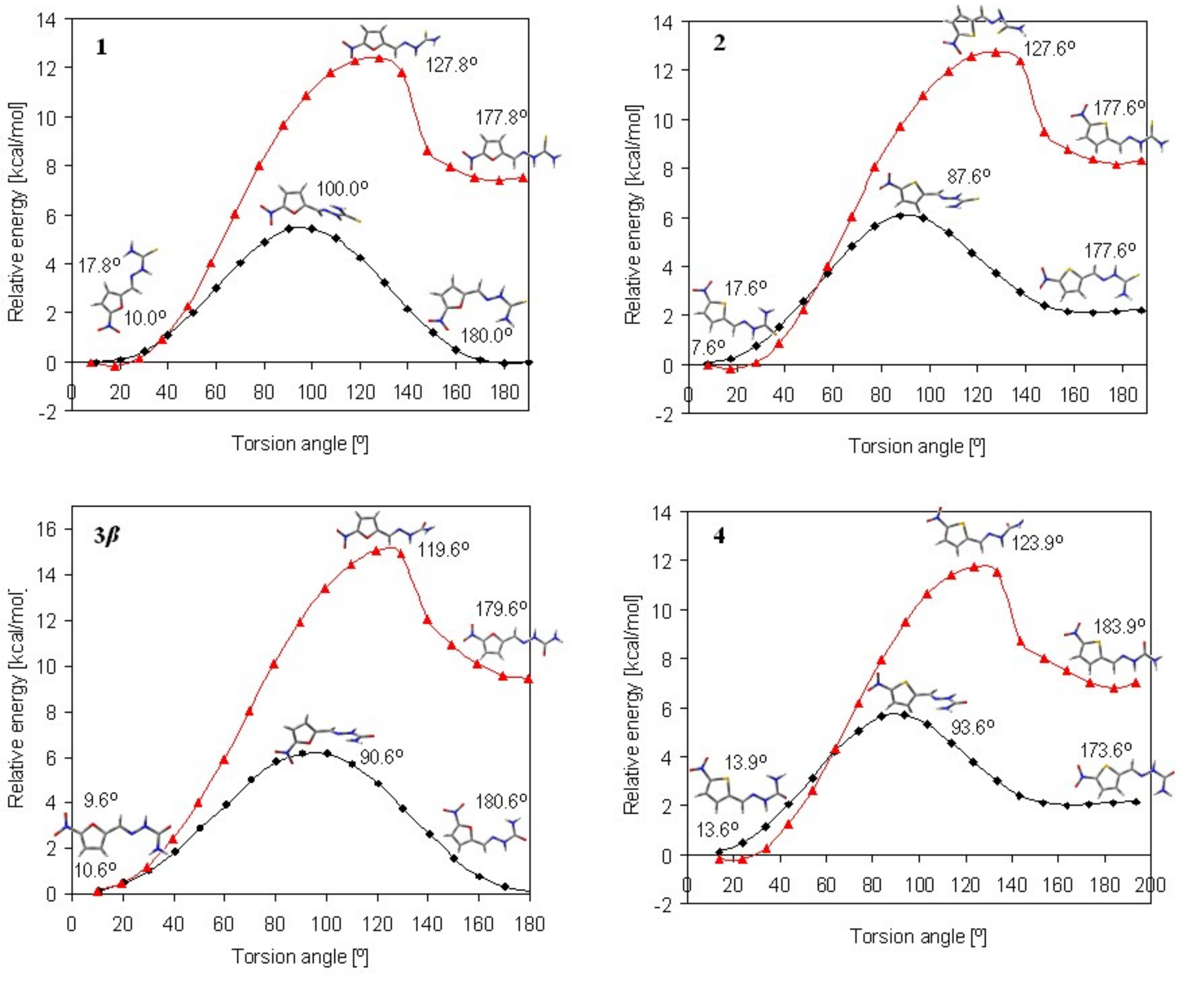

Figures S1. The energy profiles for conformers obtained by the rotation around C(imine)-C(ring) bond (black curve) and C(amide/thioamide)-N(hydrazinic) bond (red curve). The values of torsion angles existing in conformations used in calculations as a staring ones are indicated below lower left depiction of molecule in each graph for N(imine)-C(imine)-C(ring)-O/S(ring) angle and above for N(H2)-C(amide/thioamide)-N(hydrazinic)-N(imine) angle; values of respective angles after rotation of moieties are indicated near other depictions of molecules.

a)

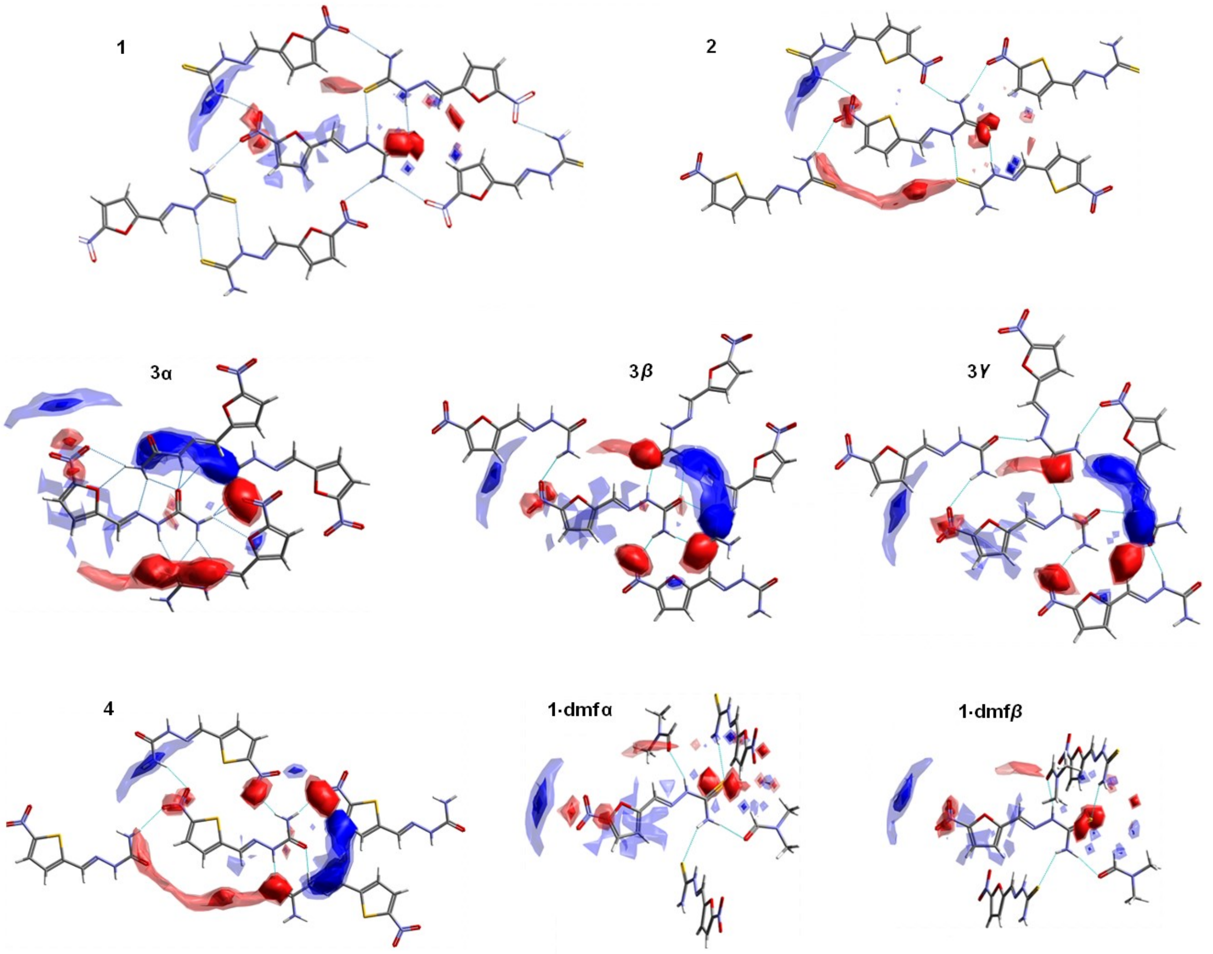

b)

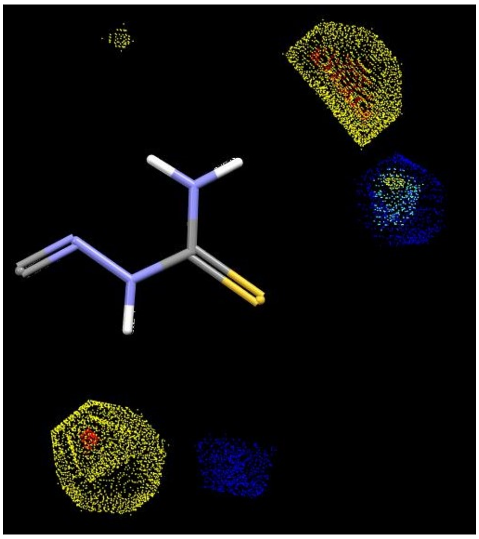

Figure S2. a) Full interaction maps with the part of crystal packing including hydrogen bond patterns. The peaks corresponding to the hydrogen bond acceptor are shown in red whereas the donor is shown in blue; b) Contoured interaction-density plot for the distribution of NH and C=S contact groups around a thiosemicarbazone group. Yellow denotes regions most preferred by the thiocarbonyl S atoms, whereas blue denotes regions most preferred by the N atoms.

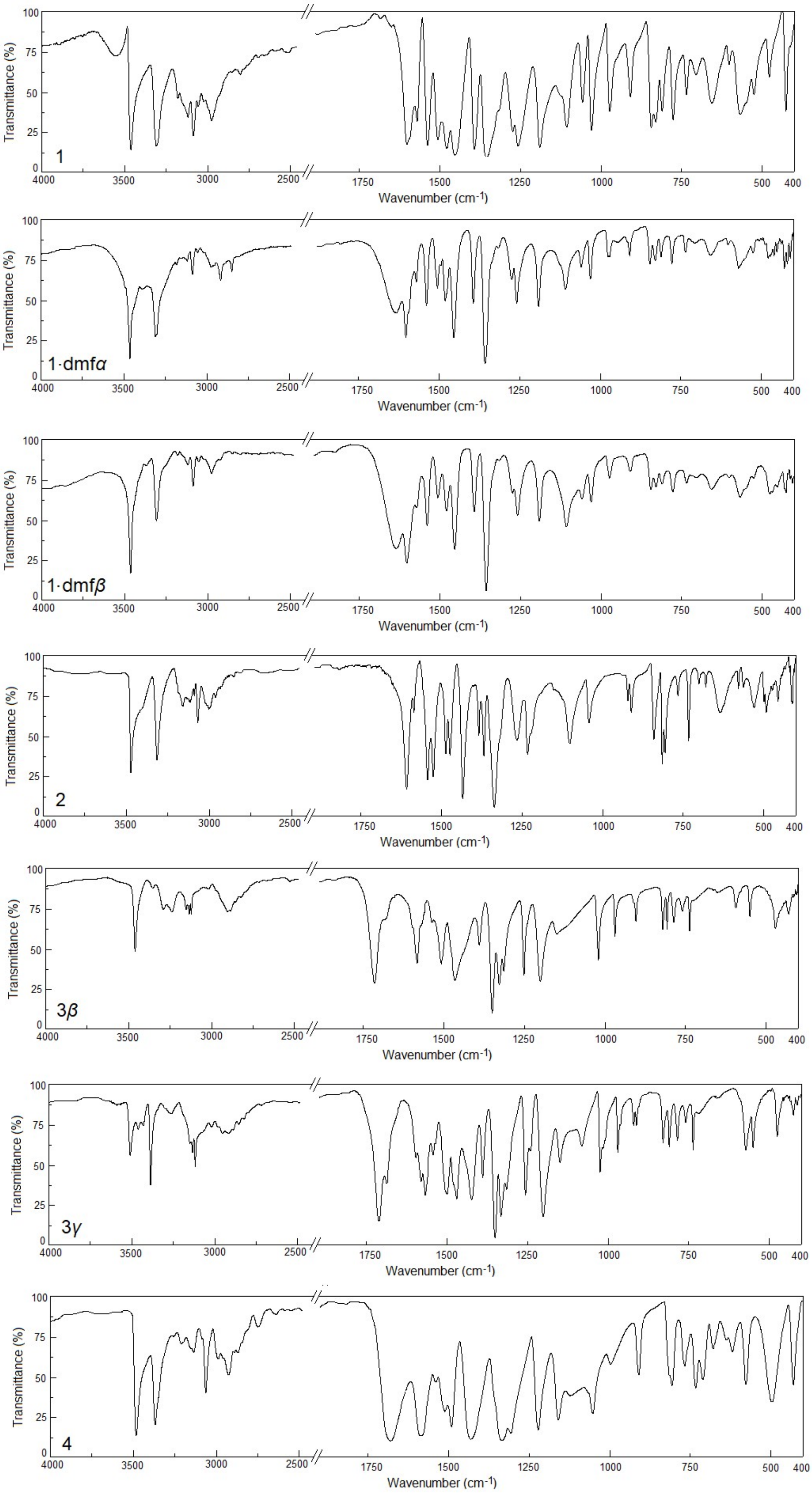

Figure S3. The IR spectra of the studied compounds.
